# Pain as a symptom of peripheral nerve sheath tumors: clinical significance and future therapeutic directions

**DOI:** 10.1186/1749-7221-3-6

**Published:** 2008-02-29

**Authors:** Michael E Sughrue, Jon Levine, Nicholas M Barbaro

**Affiliations:** 1 Department of Neurological Surgery, University of California at San Francisco, 505 Parnassus Ave, San Francisco, California, USA; 2 Department of Medicine, University of California at San Francisco, 505 Parnassus Ave, San Francisco, California, USA

## Abstract

Tumors arising from the supporting cells of peripheral nerve sheaths are relatively uncommon neoplasms, and as such many clinicians are unfamiliar with the details of their presentation, diagnosis and management. Further, little is known regarding the pathogenesis of these tumors, how they cause symptoms, and how to treat these symptoms. One classic symptom of peripheral nerve tumors is pain, however there has been little formal discussion regarding the significance of pain in this setting. Here we present a brief review of the clinical significance of pain, its relevance in pre-operative planning for the treatment of these tumors, and what is known regarding the molecular mechanisms of pain generation by these tumors.

## Epidemiology and clinical presentation of peripheral nerve tumors

Tumors arising from the supporting cells of peripheral nerve sheaths are relatively uncommon neoplasms, and as such many clinicians are unfamiliar with the details of their presentation, diagnosis and management. Further, little is known regarding the pathogenesis of these tumors, how they cause symptoms, and how to treat these symptoms.

Tumors of peripheral nerve are benign in at least 85–90% of clinically symptomatic cases, and likely a larger percentage of subclinical cases [1]. In normal patients, the majority of these tumors are histologically schwannomas, with lesser percentages made up of other benign lesions such as hemangiomas, ganglion cysts, desmoids, malignant peripheral nerve sheath tumors (MPNST's), and other malignant lesions, such as lymphoma and metastases [2]. For patients, with neurofibromatosis type 1 (NF-1), the incidence of malignancy is significantly greater: 8–10% of NF-1 patients will develop an MPNST during their lifetimes, and nearly 50% of patients with MPNST have NF-1 [3].

The typical presenting signs and symptoms of a peripheral nerve sheath tumor (PNST) involves some combination of a palpable (or radiographically visible) mass involving a peripheral nerve, loss of nerve function, and/or pain [1,3]. The etiology and significance of the first two symptoms should be relatively intuitive, for instance, the presence of a significant nerve palsy is likely due to nerve invasion and destruction by the tumor, and is highly suggestive of malignancy. However, the significance of pain in the setting of PNST is significantly less well defined, the mechanisms that cause it are more complex and poorly understood, and the proper tools to specifically or effective treat it are currently not available. A brief summary of what is currently known is the topic of the present review.

## Pain as a presenting symptom of peripheral nerve tumors

Of clear importance is the ability to differentiate between benign and malignant lesions as early as possible in the clinical work-up and management of these lesions, as they are treated very differently, and exhibit very different clinical and intraoperative behaviors. Ideally, the probable diagnosis should be known prior to surgery, as malignant tumors are more likely to require aggressive resection and possibly amputation in order to achieve any degree of oncologic control of these aggressive tumors [2,4,5]. Despite even aggressive management, the prognosis for these tumors remains poor [5-7]. Benign lesions, in contrast, are often able to be easily resected away from nerve fibers with minimum morbidity [2,4,5]. Further, resective surgery is likely to resolve or significantly improve pain in 75–85% of patients with benign tumors and pre-operative pain [2].

Current evidence suggests that in most cases, benign and malignant lesions can be differentiated pre-operatively based on clinical and radiographic characteristics. Most importantly, while either a palpable/visible mass, nerve palsy, or pain can occur in either benign or malignant tumors, all three are more common and more notable in malignant tumors. For example, rapid enlargement of a nerve mass was found in one study to predict malignant histology have a positive predictive value (PPV) of approximately 95% [3]. Also, the presence of any neurologic deficit predicts malignancy with a PPV of 73% which was identical in results published by 2 different groups [1,3]. Greater degrees of neurologic deficit (i.e. motor strength less than 3/5), appears to be exclusively a symptom of malignant tumors (PPV = 100%) [1].

Less clear is what to make of pain in the setting of a PNST, as approximately 75% of all patients with PNST (benign or malignant) have pain in some setting, and the positive predictive value of the symptom "pain" to predict malignancy is about 20–30% [1,3]. Far more important is the distinction of pain at rest versus positional pain or pain induced by pressure (i.e. the Tinel's sign). For example, one group reported that pain at rest occurred in nearly all (15/16) patients with MPNST, however only 5/99 (5%) patients with benign schwannomas or neurofibromas [1]. In contrast, 94/99 of patients with benign tumors had a positive Tinel's test [1]. Thus, further clarification of the character and timing of the pain increases the PPV of the symptom "pain" to 75%, making it a much more useful piece of information in surgical planning.

## Potiential mechanisms of pain generation in PNST and future therapeutic directions

**Figure 1:**
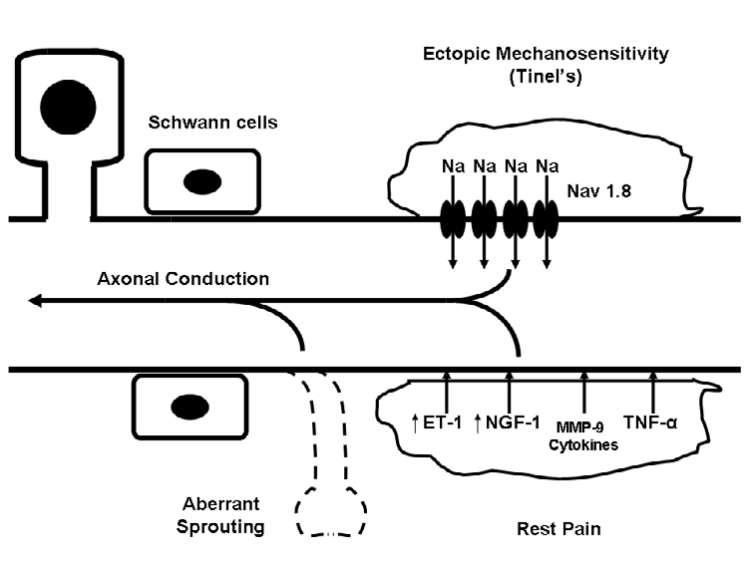
Schematic representation of possible mechanisms of algogenesis in the setting of nerve sheath tumors. Mechanisms depicted include: (A) Ectopic mechanosensitivity possibly due to increase in local concentrations of the Nav 1.4 sodium channel leading to increased axonal transmission in response to mechanical stimulation, (B) Continuous secretion of chemical algogens leading to rest pain in the absence of stimulus, (C) Aberrant axonal sprouting which fire pain stimuli constitutively.

The dichotomy seen clinically between pressure induced pain (which occurs in both benign and malignant tumors) and rest pain (largely a symptom of malignancy) suggests that these types of pain might result from distinct pathophysiologic mechanisms. It follows from this hypothesis that development of optimal therapies for each of these types of pain probably should be directed at different molecular targets. Figure 1 briefly summarizes some of the possible mechanisms involved in neuropathic pain caused by nerve tumors.

### Ectopic mechanosensitivity

The cause of pressure induced nerve pain in the setting of nerve sheath tumors is unknown. The best hypotheses formulated about the cause are extrapolations from work regarding the mechanisms of mechanosensitivity-type pain in Ad- and C-fibers seen in non-neoplastic, mechanosensitive lesions such as neuromas. Mechanosensitivity in these lesions is thought to result from progressive incorporation and buildup of a number of proteins, including mechanosensitive receptors intended for the receptor terminal of a pain receptor, into an ectopic site along an axon [8-11]. To date, the exact molecular transducer of these mechanical pain impulses is unknown.

One protein known to accumulate near areas of axonal compression, which may augment signal transduction, and thus promote the development of a mechanosensitive state, is the tetrodotoxin resistant sodium channel Nav1.8. Clinical studies have demonstrated that Nav1.8 is densely immunolocalized in the region immediately surrounding focal sites of axonal injury, such as neuromas [12-14]. Roza et al. subsequently demonstrated in vitro that Ad- and C-fibers taken from Nav1.8 null mice were markedly less prone to the development of ectopic mechanosensitivity than nerves taken from wild type mice in an experimental model of neuroma formations, and that these fibers were less likely to develop delayed spontaneous discharges in that same model [15]. The Nav1.8 is a particularly appealing therapeutic target as its expression appears to be largely restricted to peripheral nerves [15], however to date the development of a specific inhibitor has been elusive.

### Malignancy-induced nerve pain

Nerve pain caused by malignant tumors is likely chemical in nature, and results from the release of substances by malignant cells that stimulate chemoreceptive pain fibers, such as H^+^, proteolytic enzymes, cytokines and growth factors [1,16,17]. The later two classes of molecules have the particularly appealing characteristic of potentially specific inhibition as a means of alleviating cancer pain. In many cancers, invasion of the perineurium is necessary for the development of rest pain, suggesting that high periaxonal concentration of the offending algesic substance is required [18]. Given the close proximity of Schwann cells to the axon (originating inside the perineurium), it is likely that any secreted algogen is present in biologically relevant concentrations in the periaxonal space.

One potential algogen, the vasoconstrictive molecule endothelin-1 (ET-1), has been found to be released in high local concentrations in murine fibrosarcoma models of hyperalgesia [19], and was not seen in a non-sarcoma model (melanoma) [19]. Interestingly, local administration of an endothelin-A receptor antagonist significantly reduced the morphine requirement in this model [19]. Similar to fibrosarcomas, MPNST's were also found to have nearly 3-fold increased expression of the ET-1 gene compared to normal schwann cells [20], suggesting that ET-1 upregulation may be a consistent feature of sarcomas in general, and raising the possibility that ET-1 antagonism might be useful in treating MPNST rest pain, though formal *in vivo *evidence is presently lacking.

Nerve growth factor (NGF) is another algogen that has been commonly implicated as an important cause of both neuropathic and malignant cancer pain. For example, Zhu et al found that human pancreatic cancers with high levels of NGF expression demonstrated more extensive perineureal invasion, and more severe and refractory pain [18]. Other groups have demonstrated significant reduction of cancer pain with systemic NGF antagonism in murine models [16,21] While NGF may mediate these effects, in part, by inducing cancer cells to invade the perineurium, a great deal of evidence suggests that NGF may directly induce hypersensitivity in sensory neurons in both *in vitro *and *in vivo *models of neuropathic pain [22,23].

Sarcoma cells have been known for almost 50 years to produce and secrete NGF [24], and in fact, the molecule was originally discovered in experiments with sarcoma cells [25,26]. The experience with NGF expression in nerve sheath tumors is much more limited, but it seems reasonable to hypothesize that NGF might be secreted by MPNST's. More investigation is needed to further investigate this issue.

### Significance of the Schwann cell lineage to tumor-associated neuropathic pain

A large body of published work supports the notion that Schwann cells are involved in a number of dynamic interactions with their associated axons, many of which can promote (or in some cases inhibit) the development of neuropathic pain in the setting of neuronal injury. This is of special significance to the present discussion given that a significant number of peripheral nerve tumors are of Schwann cell lineage, and in theory, the disinhibition or loss of Schwann cell functions could also play a role in the production of neuropathic pain.

Schwann cells produce a number of cytokines in response to injury, many of which have been implicated in the development of neuropathic pain in the injured peripheral nerve. For example, normal Schwann cells have been found to increase expression of Tumor necrosis factor-alpha (TNF-a) in response to ex vivo compressive injury [27], and sub-endoneureal TNF-a injection in vivo induces neuropathic pain in rats [28]. Similar lines of evidence implicate Schwann cell production of matrix metalloproteinase-9 (MMP9) [29], cyclooxgenase-2 [30], and cytokines [31,32] in development of neuropathic pain.

Conversely, Schwann cells play a critical role in guidance of sprouting axons during neuronal regeneration. Regenerating neurons which lack functional Schwann cell guidance, often sprout in random directions and fail to form functional connections, which some investigators hypothesize may spontaneously fire causing neuropathic pain [33,34]. It is unclear whether this dysfunctional sprouting occurs in the setting of Schwann cell neoplasia, however it is reasonable to hypothesize that these cells likely are atleast less than ideal cellular guideposts for regenerating neurons.

While little published work has focused on the occurrence of either phenomenon in peripheral nerve tumor, either seems like a reasonable starting point for further investigation in this area.

## Conclusion

Some form of pain is seen in most patients with peripheral nerve tumor, regardless of their histopathology. However, careful delineation of the nature and character of the pain seems to provide valuable information for planning the surgical approach to these tumors. Lesions with a significant degree of rest pain should be considered as potentially malignant in terms of pre-surgical planning. Additionally, a better understanding of the chemical and molecular causes of pain in these lesions will likely lead to increased therapeutic options for palliating pain from tumors involving and invading peripheral nerves.

## Authors' contributions

MS wrote substantial portion of manuscript. JL contibuted significant portion of ideas, especially section on mechanisms of neuropathic pain. NMB concept conception, wrote significant portion of manuscript.
